# Local Strand-Breakage Detection in Multi-Strand Anchorage System Using an Impedance-Based Stress Monitoring Method—Feasibility Study

**DOI:** 10.3390/s19051054

**Published:** 2019-03-01

**Authors:** Ngoc-Loi Dang, Thanh-Canh Huynh, Jeong-Tae Kim

**Affiliations:** Department of Ocean Engineering, Pukyong National University, Busan 48723, Korea; loi.ngocdang@gmail.com (N.-L.D.); ce.huynh@gmail.com (T.-C.H.)

**Keywords:** strand breakage detection, multi-strands system, impedance-based method, impedance signature, PZT interface

## Abstract

This study investigates the feasibility of impedance-based stress monitoring method for local-strand breakage detection in multi-strand anchorage systems. Firstly, stress fields of a multi-strand anchorage system are numerically analyzed to examine anchorage’s responses sensitive to local strand breakage. Secondly, an impedance-based stress monitoring technique via the PZT interface is outlined. Thirdly, a novel hoop-type PZT interface is designed for the multi-strands anchorage to monitor the stress variation induced by the strand breakage. Local dynamic responses of the hoop-type PZT interface are analyzed to predetermine the effective frequency ranges. Finally, the numerical feasibility of the proposed method is verified on a seven-strand anchorage system under various strand breakage cases. Variations in impedance responses are statistically quantified, and broken strands are localized by linear tomography analysis of damage indices. A lab-scale experiment is also conducted on a multi-strands anchorage to evaluate the realistic performance of the hoop PZT interface for impedance-based stress monitoring method.

## 1. Introduction

Post-tensioned multi-strand anchorage systems play a key role in a wide range of civil infrastructure such as suspension bridges, cable-stayed bridges, and nuclear containment buildings. After the procedure of post-tensioning construction, the anchorage system often faces with instantaneous prestress losses that arise from the movement of strands prior to the seated wedges and elastic shortening of concrete components [1]. Due to various operational conditions and environmental effects, moreover, the post-tensioning system suffers the time-dependent prestress losses which are mostly resulted from creep and shrinkage of concrete, corrosion, and relaxation of prestressing strands. Since severe prestress loss and local strand breakage could lead to the catastrophic collapse of whole structure [2,3], the uncertain level of prestress force needs to be securely monitored during the lifetime for the structural sustainability.

Among various structural health monitoring (SHM) techniques, strain-based approaches can be regarded as simple techniques for health assessment of the tendon-anchorage systems. Kim et al. [4] embedded an optical fiber with distributed FBG (fiber Bragg grating) into a center of the seven-wire strand (the so-called ‘smart tendon’) to estimate the prestress level of bridges. Nonetheless, the high cost of the FBG-embedded tendon and interrogation system have hindered the smart tendon being widely used. Abdullah et al. [5] used an array of low-cost electrical strain gauges (ESGs) mounted on the anchor surface of the head surface to determine wire breakage in multi-strand anchorage system. Although the technique is relatively simple and cost-effective, the ESGs themselves do not have natural reference points, leading to less accuracy for long-term monitoring. Additoinally, the ESGs’ signals could be easily affected by electrical noise and environmental variation. Bartoli et al. [6] adopted acoustic emission (AE) to detect the loss of prestress force in a lab-scale multi-wire strand model. However, the usage of the AE signal is limited since it can be affected by external loads and impacts and its travel distance is dependent on the quality of transducers.

Recently, the impedance-based method has emerged for its easy applicability and high sensitivity to small incipient damage [7,8,9]. In the method, a PZT (lead zirconate titanate) sensor is surface-bonded to a host structure to measure high-frequency impedance responses which are later quantified as local damage features. Due to its promising advantages, several researchers have adopted the impedance-based method for tendon force monitoring in prestressed structures [10,11]. In their studies, PZT patches were directly attached to the anchorage zone to acquire impedance signatures. However, the direct attachment of the PZT sensor often causes the difficulty in reconfiguration of the sensor and determination of the sensitive frequency range. Nguyen et al. [12] proposed an interface washer for monitoring bolted connection. It is difficult to implement this technique into existing structures because the interface washer needs to be inserted into the contact between the bolt nut and the bearing plate. To overcome these issues, Huynh et al. [13] developed a portable PZT interface which embeds a PZT patch into a mountable structure. Numerical and experimental works demonstrated the applicability of the PZT interface technique for tendon anchorage monitoring [14,15]. 

So far, the impedance-based SHM studies have focused mostly on assessing the global prestress loss in a lab-scale tendon-anchorage system with mono-strand. For multi-strand anchorage systems, damage can occur at any local strands. Thus, the localization of locally damaged strands is needed to accurately estimate the integrity of the entire system, and this problem remains unsolved for impedance-based anchorage monitoring. Further, there is a need to develop an alternative piezoelectric interface device which is able to catch the impedance variation in multi-strands tendon anchorages.

In this study, the feasibility of impedance-based stress monitoring method for local strand-breakage detection in multi-strand anchorage system is presented. Firstly, stress fields of a multi-strand anchorage system are numerically analyzed to examine anchorage’s responses sensitive to local strand breakage. Secondly, an impedance-based stress monitoring technique via the PZT interface is outlined. Thirdly, a novel hoop-type PZT interface is designed for the multi-strands anchorage to monitor the stress variation induced by the strand breakage. Local dynamic responses of the hoop-type PZT interface are analyzed to predetermine effective frequency ranges. Finally, the numerical feasibility of the proposed method is verified on the FE model of a seven-strand anchorage system under various strand breakage cases. The stress variation on the anchor head is estimated by using the resonant frequencies of impedance signatures. Variations in impedance responses are statistically quantified, and broken strands are localized by linear tomography analysis of damage indices. A lab-scale experiment was also conducted on a multi-strands anchorage to evaluate the realistic performance of the hoop PZT interface for impedance-based stress monitoring.

## 2. Analysis of Stress Fields in a Multi-Strand Anchorage System

### 2.1. FE Model of a Multi-Strand Anchorage System

**Figure 1:**
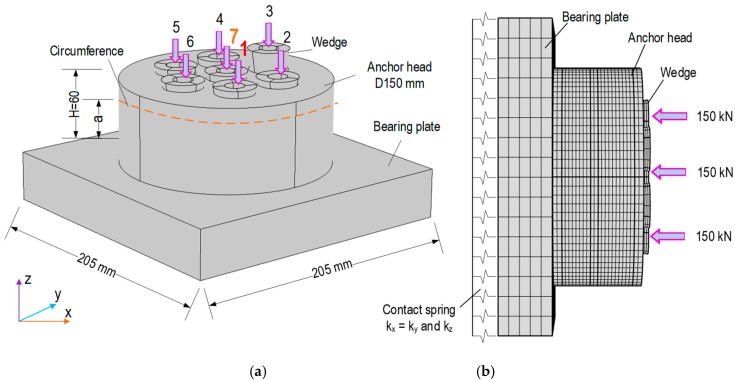
FE model of seven-strand anchorage system for stress analysis. (**a**) Multi-strand anchorage system and (**b**) discretization and boundary condition.

To analyze the stress variation induced by local strand breakage, a finite element (FE) model of a multi-strands anchorage was established using COMSOL Multiphysics (COMSOL Inc., Burlington, MA, USA). The FE model consists of a bearing plate, and seven-strand anchor head with wedges, as shown in Figure 1a. The geometric and material properties of the FE model were selected based on the post-tensioning system of VLS type E [16]. The anchorage was designed to fit with seven strands 15.2 mm (note that a *ϕ*15.2 strand has a tensile strength of 260 kN). The bearing plate has the size of 205 × 205 × 35 mm, the anchor head measures *ϕ*150 mm and 60 mm in height, and the wedge has top and bottom diameters of 29 mm and 17 mm, respectively.

As depicted in Figure 1b, the anchor head, bearing plate, and wedges were meshed by 3D solid elastic elements. The total number of meshed elements is 49,110 including 6510 elements for the bearing plate, 4536 elements for the wedges, and 58,064 elements for the anchor head. Tetrahedral elements were used for the bearing plate and the anchor head, and hexahedral elements were used for the wedges. Material properties of the steel anchorage components are listed in Table 1. As shown in Figure 1b, the remaining structure (i.e., concrete block) was simulated by the contact springs at the bottom of the bearing plate [15]. Spring constants were selected as *k_z_* = 5 × 10^15^ (N/m/m^2^), and *k_x_* = *k_y_* = 2.5 × 10^15^ (N/m/m^2^). Since the prestressing strands are anchored by wedges into the anchor head’s holes, the strand breakage causes directly local stress variation in the anchor head rather than the remaining parts of the anchorage system. In addition, the anchor head is the most rigid component, so its local stress field could be less affected by boundary components such as the concrete block. Thus, to focus on analyzing the local stress variation, the value of spring stiffness was selected to avoid the effect of boundary condition on stress variation due to the strand breakage (see Appendix A). 

The stress analysis of the FE model was conducted for the intact and two damage cases. As shown in Figure 1b, a force of 150 kN (which is equivalent to 60% of the tensile strength of the strand) was applied to each of the seven wedges (i.e., wedges 1–7) to simulate the initial prestressing condition (i.e., intact case) of the anchorage. The total prestress force applied to the anchorage was 1050 kN. As the first damage case, an outer strand (Strand 1) was removed to simulate the local breakage by reducing the applied force at wedge 1 to zero. As the second damage case, the center stand (Strand 7) was removed by assigning the applied force at the wedge 7 to zero. It is noted that a broken strand resulted in a 14% reduction of the total prestress force.

### 2.2. Variation of Stress Fields Due to Local Strand Breakage

#### 2.2.1. Effects of Local Strand Breakage on Stress Components 

**Figure 2:**
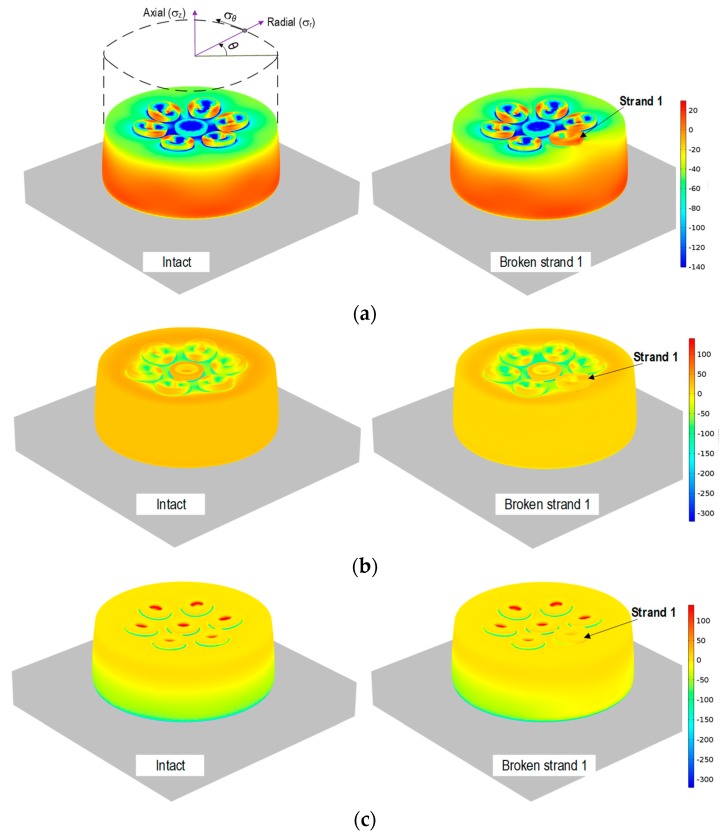
Variation of stress fields due to strand breakage. (**a**) Circumferential stress (*σ_θ_*), (**b**) radial stress (*σ_r_*), and (**c**) axial stress (*σ_z_*).

For the anchorage under local strand breakage, three stress components including circumferential, radial, and axial stresses were analyzed as indicated in Figure 2. As shown in Figure 2a, the circumferential stress (*σ_θ_*), which is the normal stress in the tangential direction of the anchor head, was uniformly distributed over the anchor head in the intact case. As Strand 1 was broken, *σ_θ_* was significantly changed near the broken strand at the top of the anchor head. As shown in Figure 2b, the radial stress (*σ_r_*), which is the stress perpendicular to and coplanar with the symmetry axis of the anchor head, was changed insignificantly before and after the breakage. As also shown in Figure 2c, the axial stress (*σ_z_*), which is the normal stress parallel to the longitudinal axis of the anchor head, was changed near the bottom of the anchor head due to the breakage of Strand 1. 

Comparatively, the breakage-induced variation of the circumferential stress was more locally concentrated near the broken strand than those of two other stresses. This result suggests that the variation of circumferential stress is suitable for detecting local strand breakage in the anchorage.

#### 2.2.2. Sensitivity of Circumferential Stress to Local Strand Breakage

**Figure 3:**
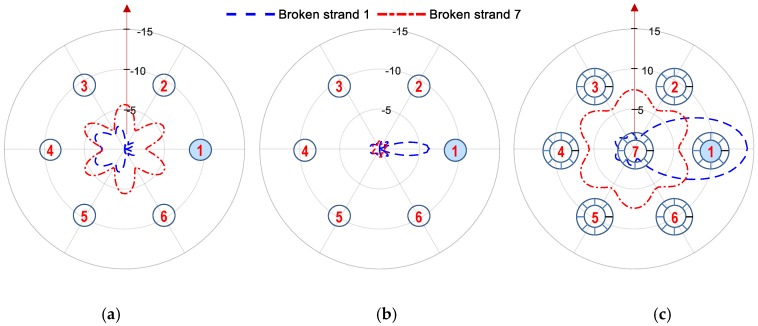
Circumferential stress variation (MPa) at relative heights of the anchor head. (**a**) Near-bottom (*a*/*H* = 0.15), (**b**) middle (*a*/*H* = 0.5), and (**c**) near-top (*a*/*H* = 0.9).

As shown in Figure 3, the sensitivity of the circumferential stress to the local strand breakage was investigated with respect to the relative height on the anchor head. For the two damage cases (i.e., Strand 1 breakage and Strand 7 breakage), three positions on the anchor head were examined: *a/H* = 0.15 (i.e., near the bottom), *a/H* = 0.5 (i.e., in the middle), and *a/H* = 0.9 (i.e., near the top). As noted in Figure 1a, *a/H* is the relative height of the examined position on the anchor head.

For the breakage of Strand 1 (i.e., an outer strand), the near-top position (*a/H* = 0.9) shows the most variation of the circumferential stress occurred close to the local breakage (see Figure 3c). Meanwhile, the near-bottom position (*a/H* = 0.15) and the middle position (*a/H* = 0.5) shows only slight variations under the same damage scenario (see Figure 3a,b). Similarly, for the breakage of Strand 7 (i.e., the center strand), the circumferential stress near the top anchor head (*a/H* = 0.9) experienced the largest variation as compared to the two other positions. The above analyses demonstrated that the near-top position produced the most sensitive circumferential stress due to the local strand breakages. Thus, the circumferential stress would have the potential to be utilized for detecting locally damaged strands in the multi-strand anchorage system.

## 3. Impedance-Based Stress Monitoring Method

### 3.1. PZT Interface for Impedance Measurement

**Figure 4:**
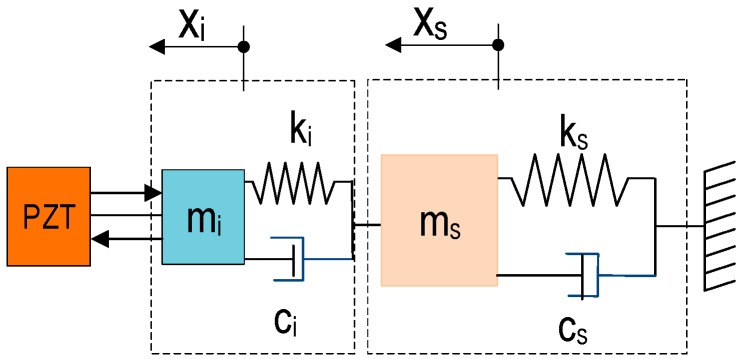
2-DOF impedance model.

In this study, an impedance-based stress monitoring technique via a PZT interface is proposed. The PZT interface is utilized to measure electro-mechanical (EM) impedance signatures in predetermined frequency bands. The PZT interface technique was developed to define the so-called ‘effective frequency bands’ for impedance monitoring practice in 10–100 kHz range [12,13,14]. In the technique, a PZT sensor is embedded in an interfacing device to acquire impedance signatures from a host structure. The coupling between the PZT interface and the host structure could be simplified as a 2-DOF (degree-of-freedom) impedance model [14], as shown in Figure 4. The governing equation of the 2-DOF system under an external harmonic excitation at a PZT-driving point, (*f_ext_*), is as follows:

(1) $$\left[\begin{array}{cc} m_{i} & 0\\ 0 & m_{s} \end{array}\right]\left\{\begin{array}{c} {\ddot{x}}_{i}\\ {\ddot{x}}_{s} \end{array}\right\}+\left[\begin{array}{cc} c_{i} & -c_{i}\\ -c_{i} & c_{i}+c_{s} \end{array}\right]\left\{\begin{array}{c} {\dot{x}}_{I}\\ {\dot{x}}_{s} \end{array}\right\}+\left[\begin{array}{cc} k_{i} & -k_{i}\\ -k_{i} & k_{i}+k_{s} \end{array}\right]\left\{\begin{array}{c} x_{i}\\ x_{s} \end{array}\right\}\;\;=\;\;\left\{\begin{array}{c} f_{ext}\\ 0 \end{array}\right\}$$

where ${\ddot{x}}_{i}$, ${\ddot{x}}_{s}$, ${\dot{x}}_{i}$, ${\dot{x}}_{s}$, $x_{i}$, $x_{s}$ are accelerations, velocities, and displacement of the interface and the host structure, respectively; *m_i_*, *c_i_*, and *k_i_* are mass, damping coefficient and stiffness of the interface, and *m_s_*, *c_s_* and *k_s_* represent for modal properties of the host structure. When $f_{ext}=\;F_{0}e^{i\omega t}$, the steady-state solution can be written as $x_{i}\;=\;X_{i0}e^{i\omega t}$, $x_{s}\;=\;X_{s0}e^{i\omega t}$. 

The coupling structural mechanical (SM) impedance of the interface and the target structure at the PZT-driving point is calculated [14], as follows:

(2) $$Z_{s}\left(\omega \right)\;\;\;=\;\;\;\frac{K_{11}\left(\omega \right)K_{22}\left(\omega \right)-K_{12}^{2}\left(\omega \right)}{i\omega K_{22}\left(\omega \right)}$$

where *ω* is input frequency sweep; dynamic stiffness coefficients are $K_{11}\left(\omega \right)\;=\;-\omega^{2}m_{i}+i\omega c_{i}+k_{i}$, $K_{22}\left(\omega \right)\;=\;-\omega^{2}m_{s}+i\omega \left(c_{i}+c_{s}\right)+\left(k_{i}+k_{s}\right)$ and $K_{12}\left(\omega \right)\;=\;-i\omega c_{i}-k_{i}$, and *i* is the imaginary unit. The EM impedance is calculated based on the output electric current, *I*(*ω*), and input harmonic voltage *V(ω)* [17]:

(3) $$Z(\omega )\;\;=\;\;\frac{V\left(\omega \right)}{I\left(\omega \right)}\;\;=\;\;{\left\{i\omega \frac{w_{p}l_{p}}{t_{p}}\left[{\bar{\varepsilon }}_{33}^{T}-\frac{Z_{a}\left(\omega \right)}{Z_{s}\left(\omega \right)+Z_{a}\left(\omega \right)}d_{31}^{2}{\bar{Y}}_{11}^{E}\right]\right\}}^{-1}$$

where *t_p_, l_p_, w_p_* are the thickness, length, and width of the PZT patch; ${\bar{\varepsilon }}_{33}^{T}\;=\;(1-i\delta )\varepsilon_{33}^{T}$ is the complex dielectric permittivity at constant stress; ${\bar{Y}}_{11}^{E}\;\;=\;\;\;(1+i\eta )Y_{11}^{E}$ is the complex Young’s modulus patch at zero electric field in the length direction; *d_3x_* is the coupling piezoelectric constant in x-direction at constant stress; *η* is damping loss coefficient; *δ* is dielectric loss coefficient of piezoelectric; and $Z_{a}\left(\omega \right)\;\;\;=\;\;\;{\hat{Y}}_{11}^{E}w_{p}t_{p}/(i\omega l_{p})$ the SM impedance of the piezoelectric patch.

Equation (3) shows that the real part of EM impedance, *Z(ω*), serves as the function of SM impedance of both PZT patch and interface-host structure system. When the mechanical and electrical properties of PZT patch are constant and no loss of energy is caused by a bonding layer between PZT patch and interface, then the SM impedance of PZT patch, *Z_a_(ω*)*,* remains constant. The SM impedance of the interface-host structure, *Z_s_(ω*), can be affected by the change of external factors such as stress and temperature variations. Hence, any changes in the host structure that lead the shift of impedance signatures can be measured via the PZT interface.

### 3.2. Impedance-Based Stress Monitoring via PZT Interface

**Figure 5:**
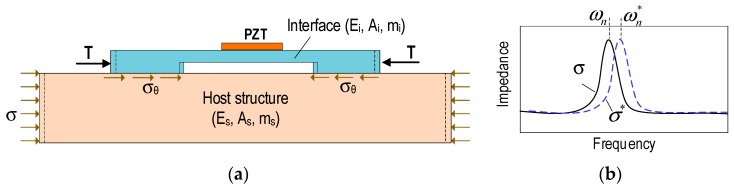
PZT interface technique for stress monitoring. (**a**) PZT interface mounted on a host structure and (**b**) shifts in EM impedance signature due to stress change.

An impedance-based stress monitoring method via PZT interface is outline. The PZT interface prototype is a beam-like structure with a PZT patch embedded in the middle [13,18]. As shown in Figure 5a, it is assumed that the PZT interface is mounted on a host structure subjected to a compressive stress *σ*. Then the compressive stress *σ* of the host structure will induce the stress *σ_θ_* and the axial force T into the PZT interface (see Figure 5a). Consequently, the change in structural properties occurred in the PZT interface turns out the variation in impedance signatures, as shown in Figure 5b.

The governing differential equation of the beam-like interface under the axial force *T* [19] as:

(4) $$\frac{\partial^{4}u\left(x,t\right)}{\partial x^{4}}-\frac{T}{E_{i}I_{i}}\frac{\partial^{2}u\left(x,t\right)}{\partial x^{2}}+\frac{m_{i}}{E_{i}I_{i}}\frac{\partial^{2}u\left(x,t\right)}{\partial t^{2}}\;\;\;\;=\;\;\;0$$

where *m_i_* is mass per unit area; *E_i_* and *I_i_* are Young’s modulus and the second momentum of the interface, respectively; *u* is transverse displacement along with the vertical axis. Assuming that the boundary condition of the interface is fixed-fixed, the natural frequencies *ω_n_* of the interface in the term of the compression force *T* can be obtained:

(5) $$\omega_{n}\;\;\;=\;\;\;C_{n}\sqrt{1+\frac{TL_{i}^{2}}{4\pi^{2}E_{i}I_{i}}\frac{C_{1}}{C_{n}}}\;\sqrt{\frac{E_{i}I_{i}}{m_{i}L_{i}^{4}}}$$

where *C_1_, C_n_* are coefficients depending on natural frequency order and can be found in [19].

Equation (5) shows that the natural frequencies of the interface decrease as the compression force *T* increase, and vice versa. It confirms that the stress variation in the host structure causes the change in the interface’s natural frequencies. 

As the host structure experiences the change of stress Δ*σ* = σ^*^ − *σ*, the force *T* in the interface also has the corresponding variation Δ*T* = T^*^ − *T*, thus leading to the shift in the natural frequencies of the interface that is $\Delta \omega_{n}{}^{2}\;\;=\;\;\;\omega_{n}^{*2}\;-\;\;\omega_{n}^{2}$. The relationship between the change of the interface force, and that of the natural frequencies can be determined, as follows:

(6) $$\;\Delta T\;\;=\;\frac{\;4\pi^{2}m_{i}L_{i}^{2}}{C_{1}C_{n}}\Delta \omega_{n}{}^{2}$$

When the interface is perfectly bonded on the host structure and it is under the uniform compression stress *σ*, the strain from the host structure is transferred to the PZT interface. Hence, the stress variation on the host structure can be estimated, as follows [18]:

(7) $$\;\Delta \sigma \;\;=\;\alpha \frac{4\pi^{2}}{C_{1}C_{n}}\frac{m_{i}L_{i}^{2}}{A_{i}}\frac{E_{s}}{E_{i}}\;\Delta \omega_{n}{}^{2}$$

where *E_s_* and *E_i_* are Young’s moduli of the host structure and the interface, respectively; *A_i_* and *m_i_* are the area and the mass of the interface, and *L_i_* is the interface length of the free section. The term α is a stress factor describing the stress transfer behavior from the host structure to the interface. When the stress field of the host structure is uniformly distributed and the stress variation perfectly transferred to the interface, the value of α becomes approximately the unity [18]. Conversely, the value of α should be larger than the unity.

Previous works show that the resonant impedance peaks represent the modal properties of the PZT-host structure at the excitation driven point [10,20]. From Equation (7), it is proved that the impedance responses vary along with the stress variation in the host structure. Thus, by monitoring the impedance change, it is possible to detect the stress variation.

## 4. Hoop-Type PZT Interface for Multi-Strand Anchorage System

### 4.1. Design of a Hoop-Type PZT Interface

**Figure 6:**
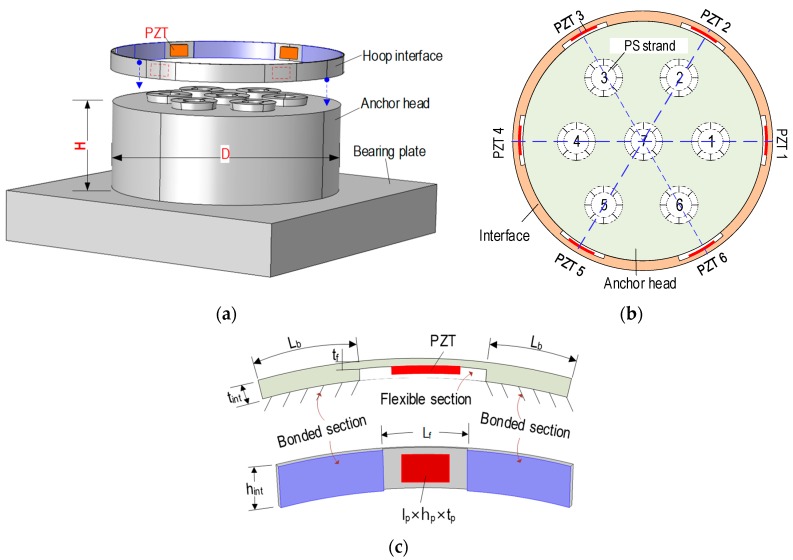
Design of a hoop-type PZTs interface for multi-strand anchorage system. (**a**) Hoop-type PZT interface on a multi-strand anchorage system, (**b**) multi-PZTs interface, and (**c**) segmental PZT interface.

As shown in Figure 6a, the multi-strand anchorage system consists of an anchor head with seven strands (i.e., six outer strands and a center strand) and a bearing plate. A hoop-type PZT interface, which fits with the anchor head, was designed to monitor the circumferential stress variation caused by the strand breakage. The concept of the portable PZT interface [13] was adopted to enhance the sensitivity of impedance signatures in predetermined frequency bands. As shown in Figure 6b, multi-PZTs (PZTs 1–6) were installed on the hoop-type interface. Each PZT sensor (which is equally distanced) was positioned close to an outer strand to sensitively monitor the health status of the strand.

As depicted in Figure 6c, a segment of the hoop interface consists of two bonded sections and a middle flexible section. Under the voltage excitation, the flexible section allows the PZT’s vibration signals while the bonded sections are used to catch the stress variation of the anchor head due to the strand breakage. Design parameters of the hoop interface are defined in Figure 6b,c. Briefly, the interface has the inner diameter *D* (i.e., the diameter of the anchor head), the thickness *t_int_*, and the height *h_int_*. The flexible section has the width *L_f_*, the thickness *t_f_*, and the height *h_int_*. The bonded section has the length of *L_b_*. The PZTs are designed with the same size, including the width *l_p_*, the height *h_p_*, and the thickness *t_p_*. A major advantage of the proposed hoop interface is the mobility that allows to be easily attached to and detached from an existing tendon anchorage.

### 4.2. Dynamic Characteristics of the Hoop-Type PZT Interface

Dynamic responses of the hoop-type PZT interface were analyzed to predetermine effective frequency ranges of impedance signatures. Based on the previous studies [14,18], the sensitive frequency range of impedance signatures coincides with the resonant responses of the PZTs interface. Thus, the local dynamics of the hoop-type interface should be analyzed to predetermine the frequency range containing resonant impedance peaks. 

#### 4.2.1. FE Model of Segmental PZT Interface

By using the anchorage system (previously described in Section 2.1) as a target structure, a hoop-type interface prototype was designed with the following geometric parameters: *D* = 150 mm, *h_int_* = 12 mm, *t_int_* = 1.5 mm, *L_f_* = 22 mm, *t_f_* = 0.5 mm distributed with the six PZT sensors. For numerical simplicity, a segment of the interface was analyzed to identify the sensitive frequency band. The segmental interface has the following parameters: *L_b_ × h_int_ × t_int_* = 30.5 × 12 × 1.5 mm for two bonded sections, *L_f_ × h_int_ × t_f_* = 22 × 12 × 0.5 mm for the flexible section, and *l_p_ × h_p_ × t_p_* = 12 × 8 × 0.267 mm for the PZT. 

For the curve-like interface body, the FRP (fiber-reinforced polymer) sheet was selected, as listed in Table 2. The material properties of the FRP were referred to [21], and the damping loss factor was selected as 2% for a bare structure [22]. As listed in Table 3, the material properties of the PZT sensor were selected for PZT 5A. 

**Figure 7:**
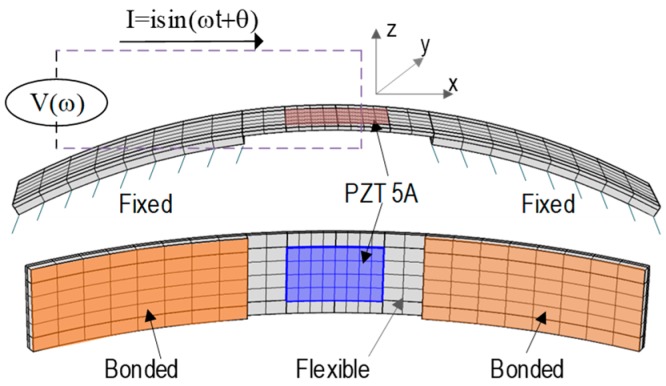
FE Model of a segmental PZT interface.

The FE model of the segmental PZT interface was established using COMSOL Multiphysics (COMSOL Inc., Burlington, MA, USA), as shown in Figure 7. The bonded sections were assigned by fixed boundaries to represent the bonding condition of the interface on the anchor head. The meshed FE model consists of 32 elements for the PZT, 264 elements for the interface body using hexahedron elements. The modules of Solid Mechanics and Piezoelectric Device, supported in COMSOL Multiphysics, were utilized to simulate eigen-frequencies and impedance analyses for the PZT interface. To obtain impedance responses, a harmonic voltage of 1 V was applied on the top surface of the PZT patch while the ground electrode was assigned to the bottom surface, as shown in Figure 7. 

#### 4.2.2. Predetermination of Sensitive Frequency Band

**Figure 8:**
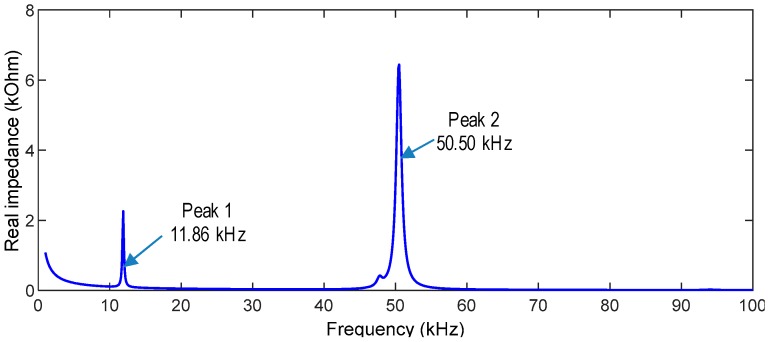
Impedance response of the PZT interface.

Impedance responses of the hoop-type PZT interface were analyzed from the 1 V voltage excitation, as shown in Figure 8. The impedance responses were simulated in the range of 1 kHz to 100 kHz (9901 intervals). From the impedance spectrum, two resonant impedance peaks were observed at 11.9 kHz (Peak 1) and 50.5 kHz (Peak 2). 

**Figure 9:**
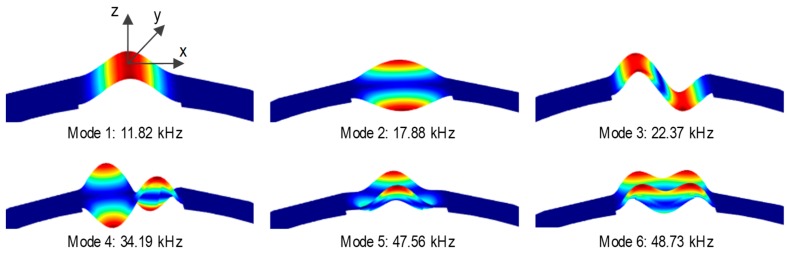
Eigen-modes of hoop-type PZT interface.

Eigen-modes of the hoop-type PZT interface were analyzed from the free vibration analysis, as shown in Figure 9. The modal analysis results revealed the first six mode shapes (Modes 1–6) of the PZT interface. Modes 1 and 3 are out-of-plane longitudinal flexural motions. Modes 2 and 4 are out-of-plane twist motions. Modes 5 and 6 are out-of-plane lateral bending motions. 

The two peak frequencies in Figure 8 were compared with the natural frequencies in Figure 9. It is found that the first resonant impedance peak (Peak 1 at 11.86 kHz) matches to the first bending mode (Mode 1 at 11.82 kHz). Also, the second resonant peak (Peak 2 at 50.50 kHz) matches to the sixth bending mode (Mode 6 at 48.73 kHz). That is, the impedance response of Peak 1 is the longitudinal bending motion with respect to the y-axis and the impedance response of Peak 2 is the lateral bending motion with respect to the x-axis. The comparative findings also suggest that the longitudinal bending motion of Peak 1 would be more sensitive to the variation of the circumferential stress than the lateral bending motion of Peak 2. These analyses can be concluded that the sensitive frequency band of the interface prototype is in the ranges from 1 kHz to 60 kHz.

## 5. Numerical Evaluation of Hoop-Type PZT Interface for Local Strand-Breakage Detection

### 5.1. FE Model of a Multi-Strand Anchorage with a Hoop-Type PZT Interface

**Figure 10:**
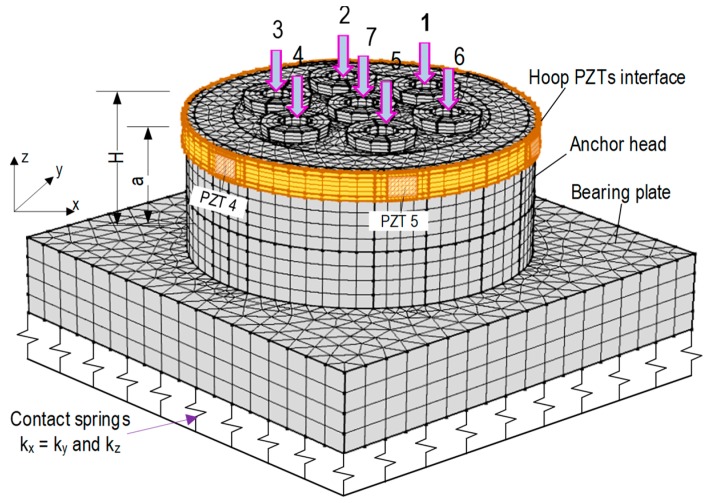
FE model of multi-strands anchorage embedded with hoop-type PZT interface.

The seven-strand anchorage system (described in Section 2.1) was utilized to evaluate the feasibility of the hoop-type PZT interface for detecting the strand breakage. As shown in Figure 6b, a hoop interface embedded with six PZTs (PZTs 1–6) was designed and circumferentially mounted on the anchor head to localize the breakage strands (i.e., Strands 1–7). The PZTs 1–6 were uniformly distributed in the hoop interface, so that they were respectively closer to Strands 1–6. As previously analyzed in Section 2.2, the circumferential stress had the most variation near-top anchor head under strand breakage, so the hoop PZTs interface was positioned at *a/H* = 0.9, as shown in Figure 10. Note that the geometric and material parameters of the hoop PZTs interface are detailed in Section 4.2.

As depicted in Figure 10, the anchorage system embedded with the hoop PZT interface was meshed using 3D block elastic elements. Specifically, hexahedron elements were used for the PZTs, the interface body, and the wedges; meanwhile, the bearing plate and the anchor head were discretized by tetrahedron elements. The meshed FE model consists of 192 elements for the PZTs, 816 elements for the interface, 3276 elements for the wedges, 14,832 elements for the anchor head, and 4520 elements for the bearing plate. 

As described previously in Section 2.1, the spring system with *k_z_* = 5 × 10^15^ (N/m/m^2^), and *k_x_* = *k_y_* = 2.5 × 10^15^ (N/m/m^2^) were assigned to the bottom of the bearing plate to represent the remaining structure of the anchorage (i.e., concrete block). The piezoelectric material properties of the PZT sensors were defined for the poling direction in their local coordinates. In order to acquire EM impedance responses from the six PZT sensors in the FE model, the global coordinate system was transformed into the local polling coordinate system for each PZT [23]. The perfectly bonded condition between the PZT interface and the anchor head was assumed for impedance simulation.

Four simulation scenarios were conducted for the FE model, as outlined in Table 4. In the intact case, a force of 150 kN was applied for all wedges (i.e., wedges 1–7) of Strands 1–7. For single damage case (i.e., Cases 1 and 2), prestress forces were removed by assigning the applied forces of wedge 1 and wedge 7 into zero. For multiple damaged strands (i.e., Case 3), Strands 1 and Strand 7 were concurrently broken and lost all prestress forces. The impedance responses were acquired in the frequency range 1–60 kHz from the six PZTs by applying a harmonic voltage of 1 V. 

### 5.2. Numerical Impedance Responses of Hoop-Type PZT Interface

**Figure 11:**
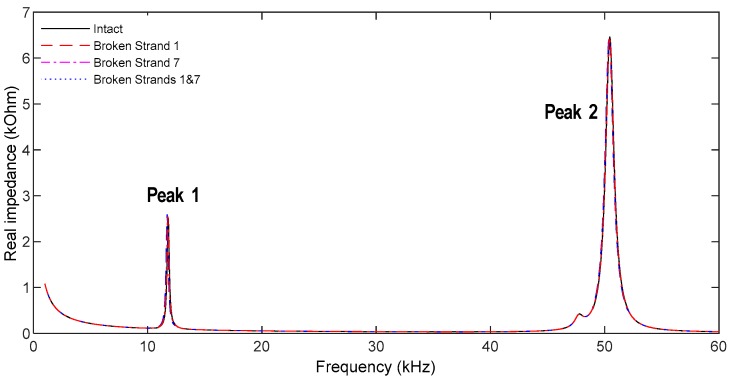
Impedance response of PZT 1 interface under strand breakage events.

As shown in Figure 11, impedance signatures of PZT 1 were plotted in the frequency range of 1–60 kHz for the intact and two damaging events (i.e., Cases 1 and 2). Within the examined frequency band, there exist two clear resonant peaks (Peaks 1 and 2), confirming the predetermined sensitive frequency band of the hoop interface (as presented in Section 4.2). It is noted that the sensitive frequency band of the proposed hoop interface was below 100 kHz, thus enabling the applicability of wireless impedance sensors [24,25,26].

**Figure 12:**
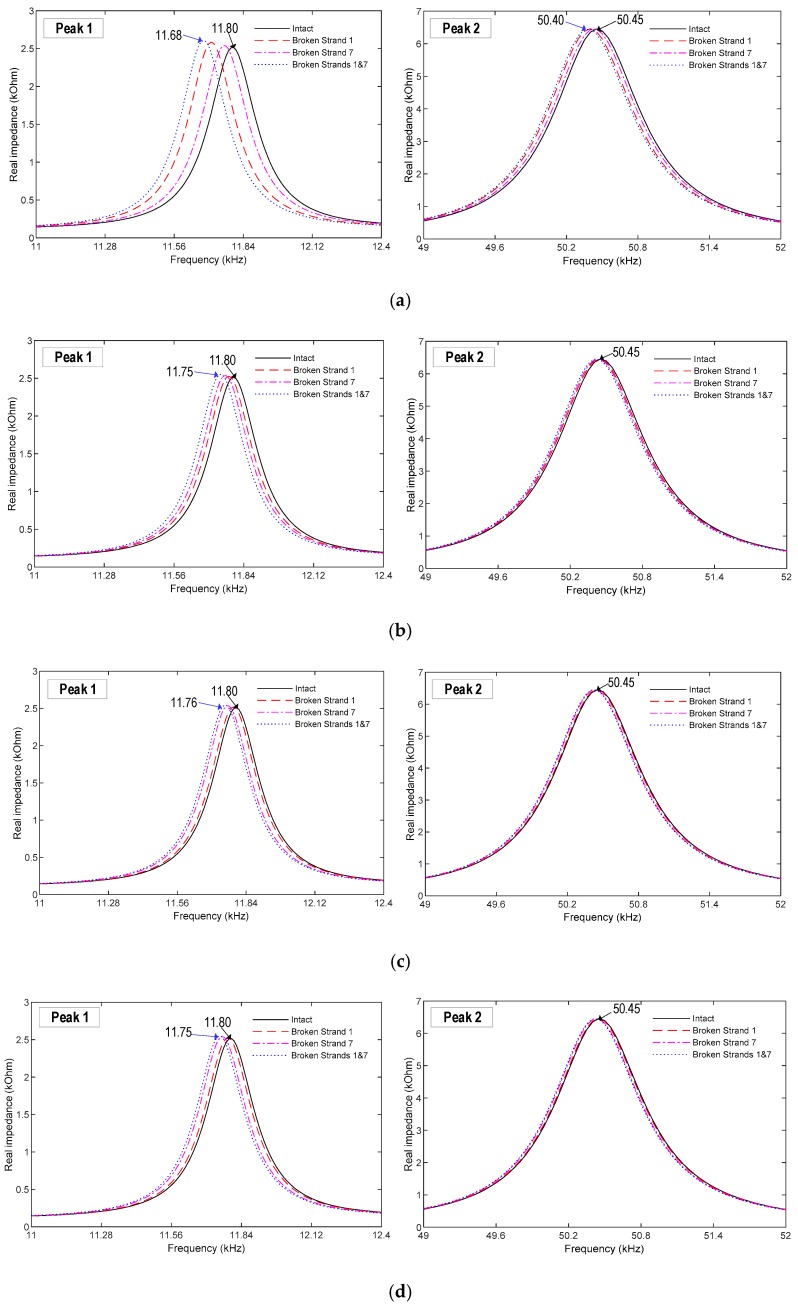

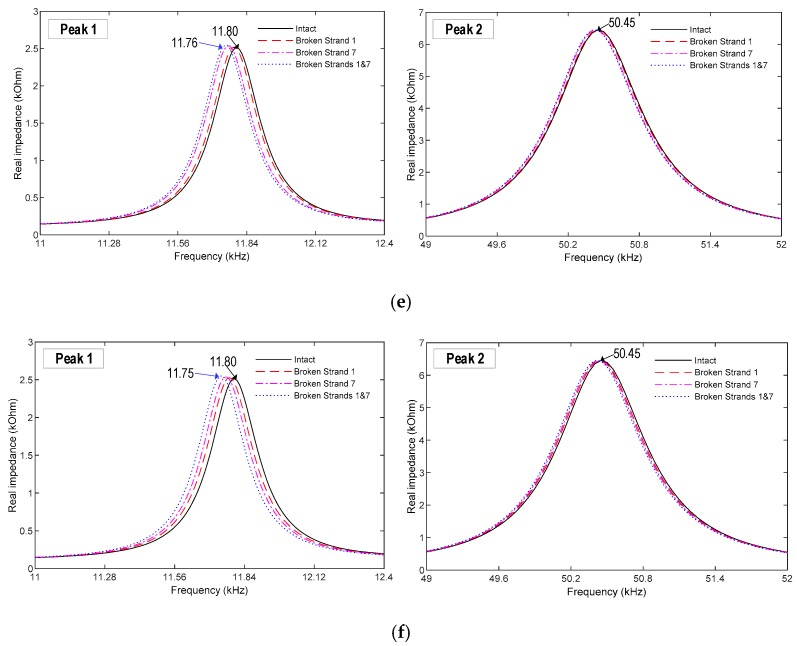
Impedance responses under strand breakage events. (**a**) PZT 1, (**b**) PZT 2, (**c**) PZT 3, (**d**) PZT 4, (**e**) PZT 5, and (**f**) PZT 6.

As shown in Figure 12, the impedance signatures of PZTs 1–6 were zoomed for 11.2–12.2 kHz and 49–52 kHz, which were the two frequency bands corresponding to the two resonant peaks (i.e., Peaks 1 and 2). It is noted that the impedance signatures of PZTs 5 and 6 are the same as those of PZTs 2 and 3, respectively, due to the symmetric positions. As observed from the figure, Peaks 1 and 2 were shifted to the left under the strand breakage events. Peak 1 shows higher sensitivity to the strand breakage than Peak 2. This result confirms that the circumferential stress mainly affected the longitudinal bending motion of Peak 1 rather than the lateral bending motion of Peak 2 (as analyzed in Section 4.2). It is also observed that PZT 1 had the highest sensitivity to the breakage of Strand 1 among the six sensors. This is because the breakage of Strand 1 mostly affected to the circumferential stress near PZT 1. Particularly, when Strands 1 and 7 were both broken, PZT 1 had 120 Hz shift for Peak 1 while other PZTs had only 40–50 Hz shifts.

### 5.3. Sensitivity of PZT Sensors’ Impedance Signatures for Locally Damaged Strands

To analyze the impedance sensitivity under the stress variation, the statistical damage index: RMSD (root mean square deviation) was used. The RMSD quantifies the change in impedance signatures [8], as follows:

(8) RMSD(Z,Z*)=(∑i=1n[Re(Z*(ωi))−Re(Z(ωi))]2)/∑i=1n[Re(Z(ωi))]2

where Re(Z(ωi)) and Re(Z*(ωi)) are the real components of the impedance signatures obtained before, and after the damage of the *i*^th^ frequency, and n denotes the number of frequency points in the swept frequency band. Ideally, the RMSD index becomes zero if there is no stress variation. Otherwise, the RMSD index will be larger than zero. 

**Figure 13:**
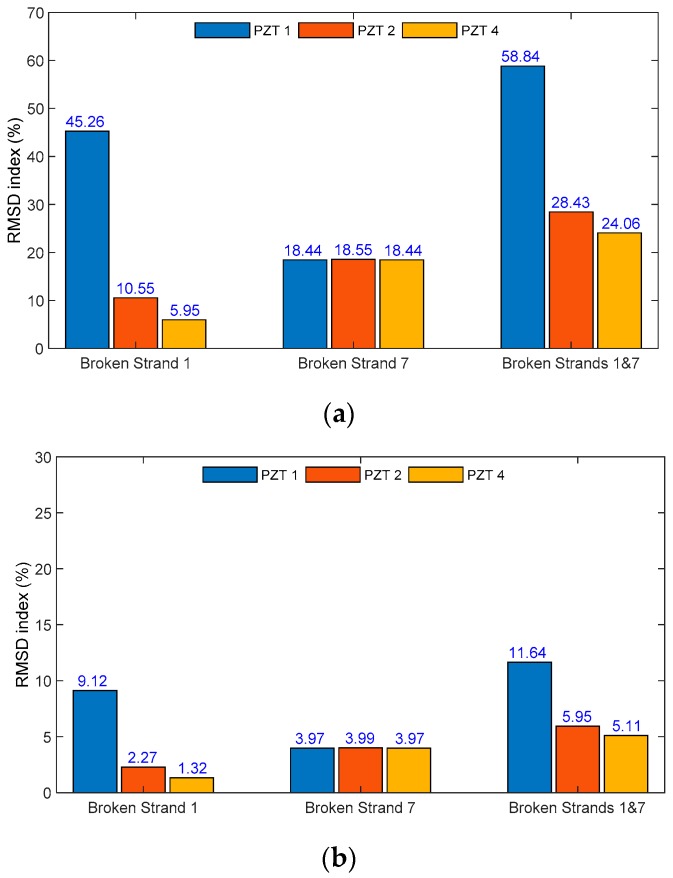
RMSD indices of PZT sensors’ impedance signature for locally damaged strand. (**a**) Peak 1’s impedance and (**b**) Peak 2’s impedance.

Figure 13 shows the RMSD damage indices under the strand breakage cases (Strand 1, Strand 7, and Strands 1 and 7) for three PZT sensors (PZT1, PZT2, and PZT4). In the Strand 1 breakage case, among three PZTs, PZT 1 was the closest to the strand breakage, while PZT 4 was the furthest, as previously described in Figure 6b. In the Strand 7 breakage case, all three PZTs were uniformly distant from the strand breakage. For Peak 1’s impedance as shown in Figure 13a, under the breakage cases of Strand 1, and Strands 1 and 7, the RMSD magnitude of PZT 1 was the largest among three PZTs. Meanwhile, under the breakage case of Strand 7, the magnitude of all PZTs was nearly the same. The observations for Peak 2’s impedance from Figure 13b show similar results. 

The above analysis confirms that the damage sensitivity of the impedance signatures is dependent on the distance from a PZT sensor to the locally damaged strand. The PZTs, which are not located close to the strand breakage location, will not give significant damage indices of impedance signatures; thus, it is difficult to identify the correct location of locally damaged strands by using only the limited number of sensors. For example, the Strand 1 breakage could not be identified if only PZT 4 and PZT 2 are used. Therefore, there exists a need to develop an alternative localization technique of locally damaged strands for multi-strand anchorage systems.

### 5.4. Linear Tomography of RMSD Index for Localization of Locally Damaged Strands

In order to localize the strand breakage, the linear tomography of RMSD index was constructed over the cross-section of the anchor head. The use of RMSD tomography can provide easy visualization of the locally damaged strands over the cross-section of the anchor head. Since the sensitivity of impedance signatures was dependent on the distance from the PZT to a locally damaged strand, the magnitude of the RMSD index would be an indicator for localizing broken strands. The RMSD index of examining frequency ranges was computed for the PZTs under strand breakage cases. For each damage cases, the linear tomography of RMSD index was established by using a polar coordinate with a linear-line graph. 

The number of PZT sensors and their positions on the interface can affect strand breakage detection results. To examine the detectability of the hoop interface with the limited number of PZT sensors, three combinations of PZTs 1–6 were investigated, as listed in Table 5. In Combination 1, all sensors: PZTs 1–6 were selected for the strand breakage detection; while PZTs 1, 3, and 5 were utilized in Combination 2, and PZTs 2, 4, and 6 were selected in Combination 3. It should be noted that in Combination 2, PZTs 1, 3, and 5 were placed respectively close to Strands 1, 3, and 5, and PZTs 2, 4, and 6 were positioned close to Strands 2, 4, and 6 in Combination 3 (see Figure 6b).

#### 5.4.1. Combination 1: All PZTs 1–6

**Figure 14:**
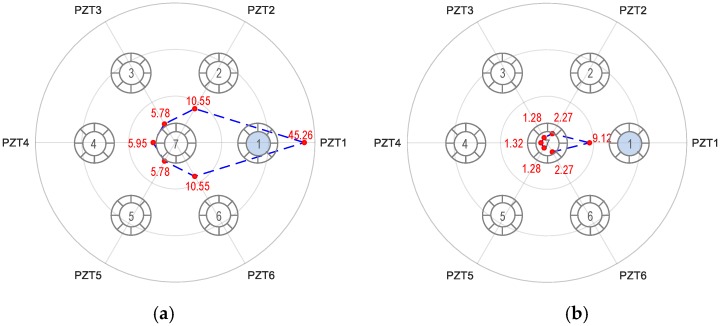
Linear tomography of RMSD indices (%) using all PZTs 1–6: Strand 1 breakage. (**a**) Peak 1’s impedance and (**b**) Peak 2’s impedance.

**Figure 15:**
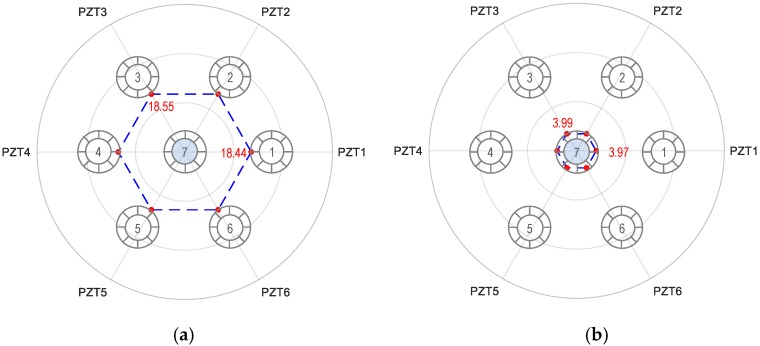
Linear tomography of RMSD indices (%) using all PZTs 1–6: Strand 7 breakage. (**a**) Peak 1’s impedance and (**b**) Peak 2’s impedance.

**Figure 16:**
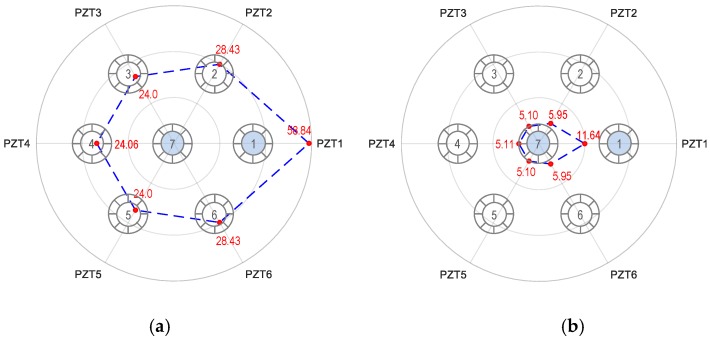
Linear tomography of RMSD indices (%) using all PZTs 1–6: Strands 1 and 7 breakage. (**a**) Peak 1’s impedance and (**b**) Peak 2’s impedance.

The RMSD indices of Peak 1’s impedance (11.2–12.2 kHz) and Peak 2’s impedance (49–52 kHz) were computed for the PZTs under three damage cases (breakage cases of Strands 1, Strand 7, and Strands 1 and 7) and plotted using linear tomography. Figure 14, Figure 15 and Figure 16 show the RMSD tomography results under Strands 1, 7 and 1 and 7 using the two impedance peaks. It is clear that the RSMD values of PZT at damaged strand were significantly different, and ignorable at the others PZTs far from strand breakage. 

Figure 14 shows the RMSD tomography results for the breakage case of Strand 1. For the first impedance peak (see Figure 14a), the RMSD at Strand 1 (i.e., 45.26%) was seven times higher than those of Strands 3–5 (i.e., 5.78%–5.95%), and four times higher than those of Strands 2 and 6 (i.e., 10.55%). The RMSD values for Peak 2 was smaller than Peak 1 but shows a similar indication of the broken strand (see Figure 14b). 

Figure 15 shows the RMSD tomography results for the breakage case of Strand 7. For the two impedance peaks, similar magnitudes of the RMSD index were found at Strands 1–6. The similar RMSD magnitudes revealed that only Strand 7 was damaged because the breakage of the middle strand (i.e., Strand 7) could cause similar variations of the RMSD index. Another possible scenario could be all of Strands 1–6 were damaged with the same severity. However, this is a rare scenario in reality. 

Figure 16 shows the RMSD tomography results for the breakage case of Strands 1 and 7. For the first impedance peak (see Figure 16a), the maximum magnitude of the RMSD was found at Strand 1, suggesting Strand 1 was damaged. However, the tomography also shows relatively significant values of the RMSD at Strands 2–6. Specifically, the RMSD value at Strand 1 (i.e., 58.84%) was about twice the values at Strands 2–6 (i.e., 24%–28.43%). This indicates that multiple strands were damaged in the anchorage. It is noted that the RMSD values at Strands 2–6 have small deviations, indicating that Strand 7 might be concurrently broken.

#### 5.4.2. Combination 2: Three PZTs 1, 3, and 5

**Figure 17:**
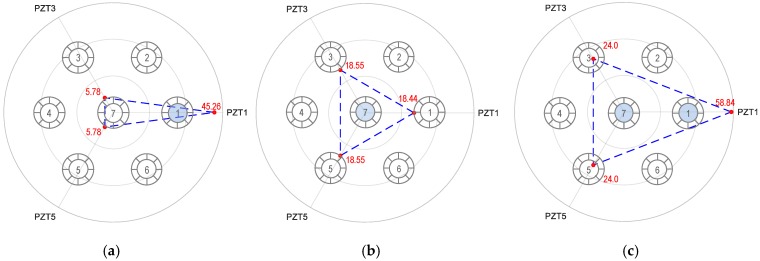
Linear tomography of RMSD indices (%) using PZTs 1, 3 and 5: Peak 1’s impedance. (**a**) Strand 1 breakage, (**b**) Strand 7 breakage, and (**c**) Strands 1 and 7 breakage.

As a potential combination of three PZTs, Figure 17 shows the RMSD tomography results using PZTs 1, 3 and 5 for the three damaged cases. Since Peak 1 significantly shifted due to the stress variation, its impedance was used for the illustration. As depicted in Figure 17a, the maximum magnitude of the RMSD value at PZT 1 was 7 times higher than that of PZTs 3 and 7, suggesting that Strand 1 was damaged. In Figure 17b, the RMSD value was almost equal for all PZTs (PZTs 1, 3, and 5), resulting from the center strand (i.e., Strand 7) damaged. Figure 17c shows the RMSD values were significant at the three PZTs. This indicates that multiple damaged strand could occur. The RMSD index was found maximum at PZT 1 (i.e., 58.84%), and the indices were equal for PZTs 3 and 5 (i.e., 24.0%). This result suggests that the potentially damaged strands could be Strands 1 and 7. 

#### 5.4.3. Combination 3: Three PZTs 2, 4, and 6

**Figure 18:**
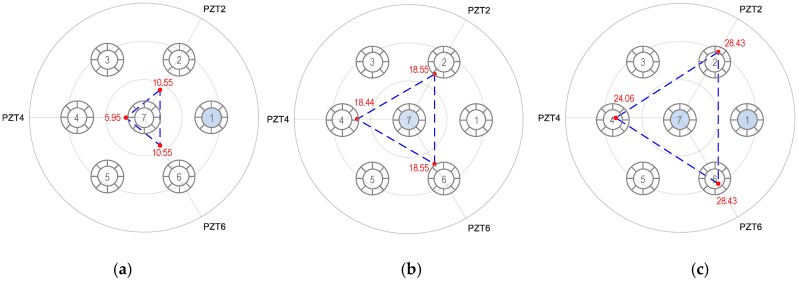
Linear tomography of RMSD indices (%) using PZTs 2, 4 and 6: Peak 1’s impedance. (**a**) Strand 1 breakage, (**b**) Strand 7 breakage, and (**c**) Strands 1 and 7 breakage.

As another possible combination of three PZTs, Figure 18 shows the RMSD tomography results using PZTs 2, 4 and 6 for the three damaged cases. As depicted in Figure 18a, the magnitude of the RMSD value was 10.55% at PZTs 2 and 6, and 5.95% at PZT 4. the RMSD magnitudes of the three PZTs were not significantly different. Because the damaged strands were far from PZTs, they resulted in less changing in the RMSD indices. The damaged strand could be Strand 1 or Strands 2 and 6 with the same severity. Figure 18b revealed that the RMSD value was nearly equal for all PZTs (PZTs 2, 4, and 6), suggesting the damaged strand was the center strand (i.e., Strand 7). As observed in Figure 18c, the RMSD values were significant and had similar values at the three PZTs, suggesting the damaged strand could be Strand 7. Although the RMSD values of PZTs 2 and 6 was slightly higher than that of PZT 1, it is difficult to make a decision on the breakage case of Strand 1. 

The above analyses suggest that the use of only three PZTs could not provide enough information to construct reliable tomography for localizing damaged strands. Conclusively, to localize all potential strand breakage cases for seven-strand anchorage system, all 6 PZTs should be designed for the hoop interface.

## 6. Experimental Feasibility of Hoop PZT Interface for Impedance-Based Stress Monitoring

### 6.1. Experimental Setup

**Figure 19:**
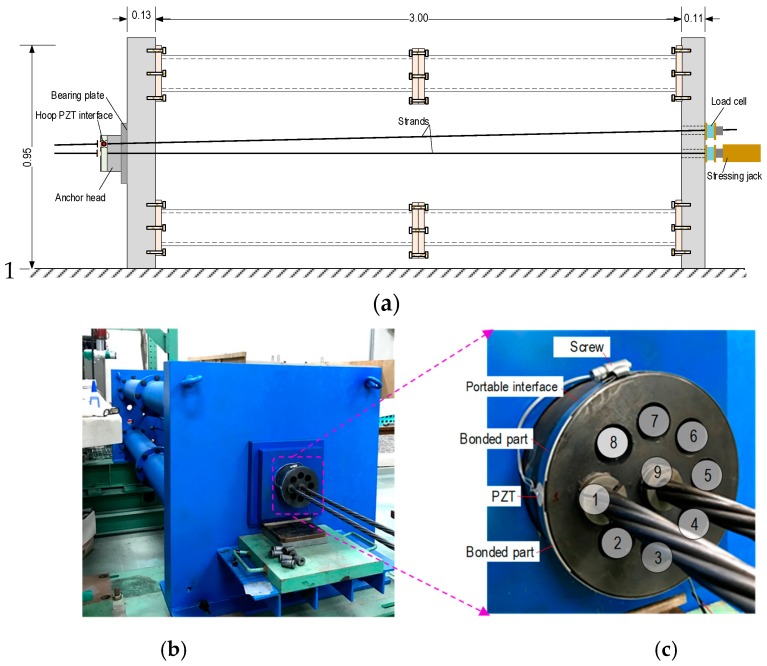
Experimental set-up of the multi-strand anchorage system. (**a**) Overview of the test setup (unit: m), (**b**) prestressed multi-strand anchorage system, and (**c**) hoop PZT interface mounted on the anchor head.

To evaluate the realistic performance of the hoop PZT interface for the impedance-based stress monitoring, a lab-scale experiment on a multi-strands anchorage was conducted, as shown in Figure 19. The anchor system consists of a bearing plate and a multi-strands anchor head with wedges which were supported by a steel frame, as shown in Figure 19a,b. For the feasibility evaluation, two strands were installed into the anchorage system. Each strand is composed of seven steel wires with *ϕ*15.2 mm and tensile strength of 260 kN. The two strands were anchored at the left ends and tensioned from the right ends using hydraulic jacks. Load cells installed at the right ends of the strands were used to monitor inflicted prestress forces.

Figure 19c shows a prototype of the hoop PZT interface mounted on the multi-strands anchor head. The hoop interface was made up steel and was clamped into the anchor head by using a screw device. For the feasibility evaluation, the prototype was designed with only one segmental PZT interface. As previously described in Figure 6, the dimensional parameters of the segmental interface were selected as: *D* = 159 mm, *L_b_ × h_int_ × t_int_* = 42 × 13.70 × 1.70 mm for the bonded sections, and *L_f_ × h_int_ × t_f_* = 20 × 13.70 × 0.7 mm for the flexible section. A circular-type PZT sensor with *ϕ*15 mm and *t_p_* = 0.20 mm was mounted in the middle of the interface by using the high strength adhesive (Loctile 401). The hoop PZT interface was installed at the near-top anchor head (*a/H* ≈ 0.9) (see Figure 19c), so that the segmental PZT interface was positioned close to Strand 1. 

Prestressing test cases for the lab-scale anchorage system is listed in Table 6. In the first four prestressing cases (i.e., PS1-PS4), Strand 9 (the center strand) was gradually tensioned from 0 to 147.2 kN with an interval of 49.1 kN. In the later cases (i.e., PS5-PS7), the prestress force of Strand 9 remained constant while that of Strand 1 (the outer strand) was gradually tensioned up to 147.2 kN with the interval of 49.1 kN. For each prestressing case, a harmonic voltage of 5 V was used to excite the PZT sensor to measure its impedance responses. HIOKI-3532 was utilized to acquire impedance signatures in 5–29 kHz range (with 0.05 kHz frequency interval). For each test case, four ensembles of the impedance signature were recorded. In order to minimize the effect of temperature variation on the impedance responses, the room temperature was kept near 13 °C by using four air conditioners.

### 6.2. Impedance-Based Stress Monitoring Result

**Figure 20:**
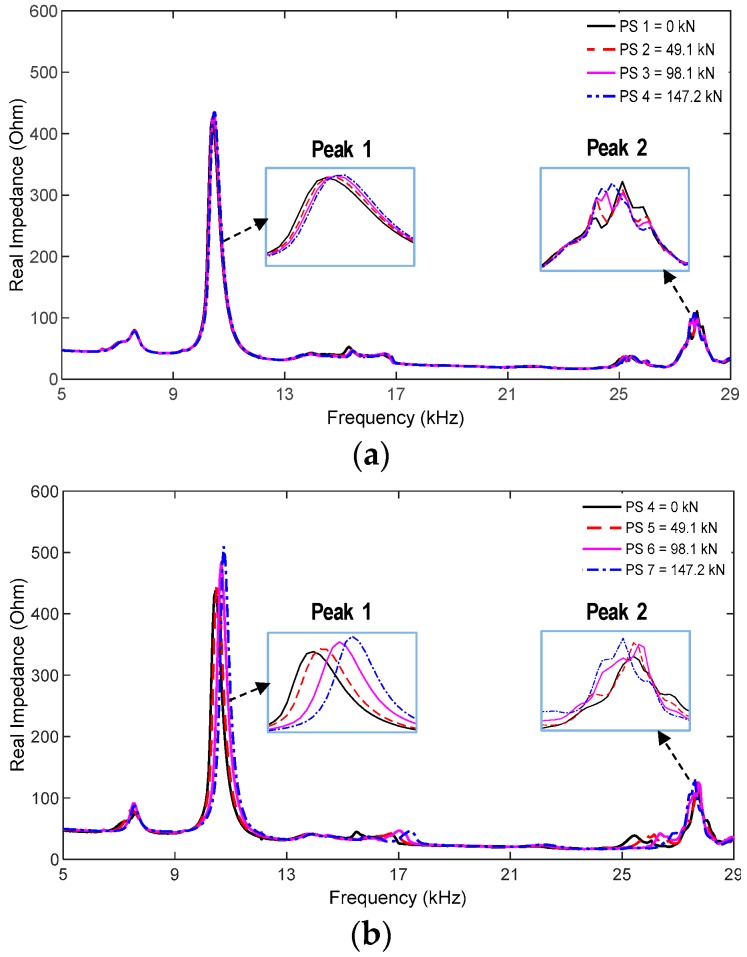
Impedance responses of the hoop PZT interface under prestressing cases. (**a**) Prestressing cases of Strand 9: PS1–PS4 and (**b**) prestressing cases of Strand 1: PS4–PS7.

Figure 20 shows the measured impedance responses of the hoop PZT interface under seven prestressing cases (i.e., PS1–PS7). Two distinct impedance peaks at 10.5 kHz and 28.1 kHz were observed within the frequency range of 5–29 kHz. The two impedance peaks were slightly varied under the prestressing cases of Strand 9 (see Figure 20a) but significantly varied under the prestressing cases of Strand 1 (see Figure 20b). This is because the PZT was positioned close to Strand 1.

**Figure 21:**
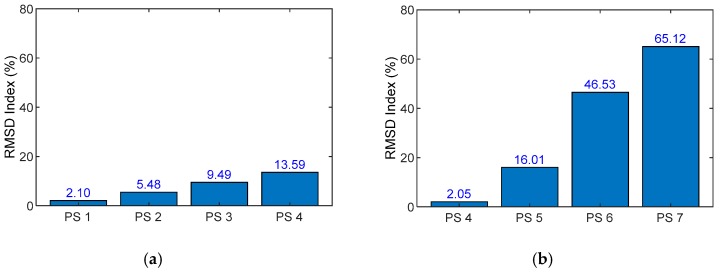
RMSD quantification of impedance responses under prestressing cases. (**a**) Prestressing cases of Strand 9: PS1–PS4 and (**b**) prestressing cases of Strand 1: PS4–PS7.

Next, the RMSD index was computed to monitor the stress variation in the anchor head under the prestressing cases. The entire frequency range of 5–29 kHz was used for the computation. Figure 21a,b shows the RMSD indices for the prestressing cases of Strand 9 (PS1–PS4) and Strand 1 (PS4–PS7), respectively. It is observed that the magnitude of RMSD indices was linearly increased with the increased prestress forces. Since Strand 1 was closer to the PZT than Strand 9, the RMSD values of Strand 1 changed more significantly than those of Strand 9 under the prestressing effects.

These experimental observations are consistent with the previous simulation results. The experimental results confirm that the impedance signature measured via the hoop PZT interface was sensitive to prestress force variations. Additionally, the sensitivity of impedance responses was dependent on the distance from the PZT to a locally damaged strand.

## 7. Conclusions

In this study, the feasibility of impedance-based stress monitoring method for local strand-breakage detection in multi-strand anchorage systems was presented. Firstly, stress fields of the multi-strand anchorage system were numerically analyzed to examine anchorage’s responses sensitive to local strand breakage. Secondly, an impedance-based stress monitoring technique via the PZT interface was outlined. Thirdly, the novel hoop-type PZT interface was designed for the multi-strands anchorage to monitor stress variations induced by the strand breakage. Local dynamic responses of the hoop-type PZT interface were analyzed to predetermine the effective frequency ranges. Finally, the numerical feasibility of the proposed method was verified on the seven-strand anchorage system under various strand breakage cases. Variations in impedance responses were statistically quantified, and broken strands were localized by linear tomography analysis of damage indices. A lab-scale experiment was also conducted on a multi-strand anchorage system to evaluate the realistic performance of the hoop PZT interface for impedance-based stress monitoring.

From the numerical and experimental analyses, at least three concluding remarks can be drawn, as follows: (1) the impedance signatures of the proposed hoop interface were sensitive to the variation in the circumferential stress induced by the strand breakage; (2) the insufficient number of PZTs on the hoop interface could lead to inaccurate localization of locally damaged strands; and (3) the feasibility of the impedance-based stress monitoring method for local strand-breakage detection in multi-strand anchorage systems was evaluated.

While most of previous studies have focused on impedance monitoring of tendon-anchorage systems with mono-strand, this study developed the hoop interface with multiple PZTs to monitor stress-induced impedance variations in the multi-strand anchorage system. Thus, the developed interface opens up a new approach using the impedance technique combined with the linear tomography analysis to detect and localize local strand breakage in multi-strands tendon anchorage. Despite some promising results, future studies remain. There is a need to conduct a full-scale experimental evaluation of the proposed method. Additionally, the FE modeling technique should be improved to better simulate the contact condition between the PZT interface and the anchor head.

## Figures and Tables

**Table 1:** Material properties of anchorage components.

Parameters	Anchor Head, Bearing Plate, and Wedges
Young’s modulus, *E* (GPa)	200
Poisson’s ratio, *υ*	0.3
Mass density, *ρ* (kg/m^3^)	7850

**Table 2:** Material properties of FRP sheet.

Young’s Modulus *E*, (GPa)	Poisson’s Ratio *ν*	Mass Density *ρ* (kg/m^3^)	Damping Loss Factor *η*
145	0.30	1700	0.02

**Table 3:** Material properties of PZT 5A.

Young’s Modulus *E*, (GPa)	Mass Density *ρ* (kg/m^3^)	Damping Loss Factor *η*	Dielectric Constant, *ε^T^*_33_ (Farad/m)	Dielectric Loss Factor *δ*	Coupling Constant **d_31_ (m/V)
62.1	7750	0.0125	1.53 × 10^-8^	0.015	−1.71 × 10^−10^

**Table 4:** Simulation cases of strand breakage in the FE model.

Case	Simulation Scenario
Intact	All wedges (Strands 1–7) were assigned by 150 kN
Case 1	Strand 1 was broken
Case 2	Strand 7 was broken
Case 3	Strands 1 and 7 were both broken

**Table 5:** Combination of PZT sensors for linear tomography of RMSD index.

Combination	PZT 1	PZT 2	PZT 3	PZT 4	PZT 5	PZT 6
1	✓	✓	✓	✓	✓	✓
2	✓		✓			✓
3		✓		✓		✓

✓: PZT sensor is selected for a combination.

**Table 6:** Prestressing cases of the lab-scale multi-strand anchorage system.

Case	Applied Prestress Force (kN)	
	Strand 9	Strand 1
PS 1	0	0
PS 2	49.1	0
PS 3	98.1	0
PS 4	147.2	0
PS 5	147.2	49.1
PS 6	147.2	98.1
PS 7	147.2	147.2

**Figure A1:**
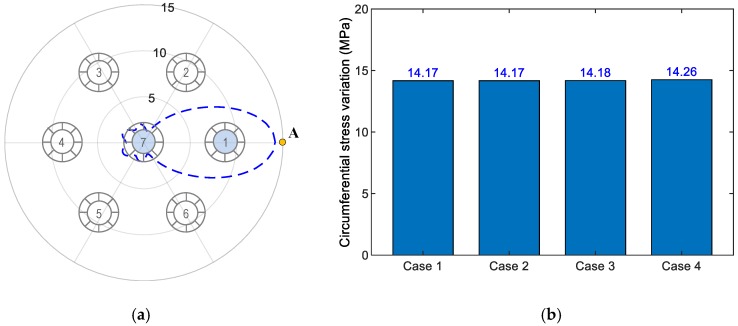
The effect of boundary condition on circumferential stress variation. (**a**) Circumferential stress variation in Case 1 and (**b**) comparison of circumferential stress variation.

## Appendix A. Selection of Spring Constants for Simulating a Concrete Block

The contact springs were used to simulate the remaining structure (i.e., concrete block). Since the anchor head is the most rigid component in the anchorage system, its local stress field could be less affected by boundary components such as the concrete block. Thus, to focus on analyzing the local stress variation, the value of spring constants was selected, so that the effect of boundary condition on the stress variation can be ignored. Four cases (i.e., Cases 1–4) of spring constants were analyzed under Strand 1 breakage. The following four cases of spring constants were assigned to the bottom of the bearing plate: (1) *k_x_* = *k_y_* = 2.5 × 10^16^ (N/m/m^2^) and *k_z_* = 5 × 10^16^ (N/m/m^2^) in Case 1; (2) *k_x_* = *k_y_* = 2.5 × 10^15^ (N/m/m^2^) and *k_z_* = 5 × 10^15^ (N/m/m^2^) in Case 2; (3) *k_x_* = *k_y_* = 2.5 × 10^14^ (N/m/m^2^) and *k_z_* = 5 × 10^14^ (N/m/m^2^) in Case 3; (4) *k_x_* = *k_y_* = 2.5 × 10^13^ (N/m/m^2^) and *k_z_* = 5 × 10^13^ (N/m/m^2^) in Case 4.

Figure A1a shows the circumferential stress variation in the anchor head under Strand 1 breakage (i.e., *a/H* = 0.9) in Case 1. The maximum magnitude was 14.17 MPa at point A, which was closest to Strand 1. Figure A1b shows the comparison of stress variations at point A for different spring constants. It is observed that the spring constants had ignorable effects on the circumferential stress variation. Particularly, in Cases 1 and 2, the circumferential stress variation remained unchanged. Therefore, to avoid the effect of the boundary condition on the local stress field of the anchor head under broken strands, the spring constants with *k_x_* = *k_y_* = 2.5 × 10^15^ (N/m/m^2^) and *k_z_* = 5 × 10^15^ (N/m/m^2^) were selected to simulate the concrete block.
